# Supplementary material for: Paramagnetic resonance in spin-polarized disordered Bose-Einstein condensates
Source: Sci Rep. 2017 May 18;7:2076. doi: 10.1038/s41598-017-01125-4 (PMC5437097; doi:10.1038/s41598-017-01125-4)
Supplement: Supplementary file 1 — Supplementary Material for “Paramagnetic resonance in spin-polarized disordered Bose-Einstein condensates” [file 41598_2017_1125_MOESM1_ESM.pdf]

Supplementary Material for “Paramagnetic resonance in spin-polarized disordered Bose-Einstein condensates”

V. M. Kovalev[1,2,3*] and I. G. Savenko[1,4,5]

[1]Center for Theoretical Physics of Complex Systems, Institute for Basic Science, Daejeon, South Korea
[2]Institute of Semiconductor Physics, Siberian Branch of Russian Academy of Sciences, Novosibirsk 630090, Russia
[3]Department of Applied and Theoretical Physics, Novosibirsk State Technical University, Novosibirsk 630073, Russia
[4]National Research University of Information Technologies, Mechanics and Optics, St. Petersburg 197101, Russia
[5]Nonlinear Physics Centre, Research School of Physics and Engineering, The Australian National University, Canberra ACT 2601, Australia
*vadimkovalev@isp.nsc.ru

ABSTRACT

In present Supplementary Material we present derivation of Eq. (7) of the main text.

Derivations

We start from Eqs. (4),

$$\begin{aligned}&\left(i\partial_t-\hat{E}_{\mathbf{p}}+\mu-U_0|\psi_+|^2-u(\mathbf{r})-\mathscr{F}\right)\psi_+=\alpha\hat{p}_-^2\psi_-,\\&\left(i\partial_t-\hat{E}_{\mathbf{p}}+\mu-U_2|\psi_+|^2-u(\mathbf{r})+\mathscr{F}\right)\psi_-=\alpha\hat{p}_+^2\psi_+.\end{aligned}\tag{1}$$

Substituting here

$$\begin{pmatrix}\psi_+(\mathbf{r},t)\\ \psi_-(\mathbf{r},t)\end{pmatrix}\to\begin{pmatrix}\psi_0(\mathbf{r})+\delta\psi_+(\mathbf{r},t)\\ \delta\psi_-(\mathbf{r},t)\end{pmatrix},\tag{2}$$

we have

$$\begin{aligned}&\left(i\partial_t-\hat{E}_{\mathbf{p}}+\mu-U_0|\psi_0(\mathbf{r})+\delta\psi_+(\mathbf{r},t)|^2-u(\mathbf{r})-\mathscr{F}\right)(\psi_0(\mathbf{r})+\delta\psi_+(\mathbf{r},t))=\alpha\hat{p}_-^2\delta\psi_-(\mathbf{r},t),\\&\left(i\partial_t-\hat{E}_{\mathbf{p}}+\mu-U_2|\psi_0(\mathbf{r})+\delta\psi_+(\mathbf{r},t)|^2-u(\mathbf{r})+\mathscr{F}\right)\delta\psi_-(\mathbf{r},t)=\alpha\hat{p}_+^2(\psi_0(\mathbf{r})+\delta\psi_+(\mathbf{r},t)).\end{aligned}\tag{3}$$

Assuming that perturbed parts of EP wave functions are proportional to external perturbation $\delta\psi_+\sim\delta\psi_-\sim\mathscr{F}$, we keep only the zero and first order corrections coming from nonlinear interacting terms in Hamiltonian:

$$\begin{aligned}&|\psi_0(\mathbf{r})+\delta\psi_+(\mathbf{r},t)|^2(\psi_0(\mathbf{r})+\delta\psi_+(\mathbf{r},t))\approx|\psi_0(\mathbf{r})|^3+|\psi_0(\mathbf{r})|^2\left(2\delta\psi_+(\mathbf{r},t)+\delta\psi_+^*(\mathbf{r},t)\right),\\&|\psi_0(\mathbf{r})+\delta\psi_+(\mathbf{r},t)|^2\delta\psi_-(\mathbf{r},t)\approx|\psi_0(\mathbf{r})|^2\delta\psi_-(\mathbf{r},t).\end{aligned}\tag{4}$$

The terms describing the interaction with external potential, $\mathscr{F}$, are simplified similarly, disregarding the $\mathscr{F}^2$ corrections:

$$\begin{aligned}&\mathscr{F}(\psi_0(\mathbf{r})+\delta\psi_+(\mathbf{r},t))\to\psi_0(\mathbf{r})\mathscr{F},\\&\mathscr{F}\delta\psi_-(\mathbf{r},t)\to 0.\end{aligned}\tag{5}$$

In term $\alpha\hat{p}_+^2(\psi_0(\mathbf{r})+\delta\psi_+(\mathbf{r},t))$ one can disregard the first term, $\alpha\hat{p}_+^2\psi_0(\mathbf{r})$, since it equals zero in the equilibrium case, $\psi_0=\sqrt{n_c}$, and it has a small value in the case when we account for the disorder.

Finally, we come up with the system of equations presented in the main text, where zero-order correction to the EP wave function satisfies the equation:

$$[\hat{E}_{\mathbf{p}}-\mu+U_0|\psi_0(\mathbf{r})|^2+u(\mathbf{r})]\psi_0(\mathbf{r})=0,\tag{6}$$

and the first order corrections are described by equations

$$\begin{aligned}
&\left(i\partial_t - \hat{E}_{\mathbf{p}} + \mu - 2U_0|\psi_0(\mathbf{r})|^2 - u(\mathbf{r})\right)\delta\psi_+(\mathbf{r},t) - U_0|\psi_0(\mathbf{r})|^2\delta\psi_+^*(\mathbf{r},t) - \alpha\hat{p}_-^2\delta\psi_-(\mathbf{r},t) = \psi_0(\mathbf{r})\mathscr{F}(\mathbf{r},t), \\
&\left(i\partial_t - \hat{E}_{\mathbf{p}} + \mu - U_2|\psi_0(\mathbf{r})|^2 - u(\mathbf{r})\right)\delta\psi_-(\mathbf{r},t) - \alpha\hat{p}_+^2\delta\psi_+(\mathbf{r},t) = 0.
\end{aligned} \tag{7}$$

Repeating the same calculation steps with the complex conjugate functions, $\delta\psi_+^*,\ \delta\psi_-^*$, yields:

$$\begin{aligned}
&\hat{G}^{-1}\begin{pmatrix}\delta\psi_+ \\ \delta\psi_+^*\end{pmatrix} - \hat{K}\begin{pmatrix}\delta\psi_- \\ \delta\psi_-^*\end{pmatrix} = \psi_0(\mathbf{r})\mathscr{F}(\mathbf{r},t)\begin{pmatrix}1 \\ 1\end{pmatrix}, \\
&\hat{\mathfrak{G}}^{-1}\begin{pmatrix}\delta\psi_- \\ \delta\psi_-^*\end{pmatrix} - \hat{K}^*\begin{pmatrix}\delta\psi_+ \\ \delta\psi_+^*\end{pmatrix} = 0,\ \hat{K} = \begin{pmatrix}\alpha\hat{p}_-^2 & 0 \\ 0 & \alpha\hat{p}_+^2\end{pmatrix},
\end{aligned} \tag{8}$$

where the Green's functions read:

$$\begin{aligned}
&\hat{\mathfrak{G}}^{-1}(\mathbf{r},\mathbf{r}';t-t') = \begin{pmatrix} i\partial_t - \frac{\hat{\mathbf{p}}^2}{2m} + \mu - u(\mathbf{r}) - (U_0 - 2U_1)|\psi_0(\mathbf{r})|^2 & 0 \\ 0 & -i\partial_t - \frac{\hat{\mathbf{p}}^2}{2m} + \mu - u(\mathbf{r}) - (U_0 - 2U_1)|\psi_0(\mathbf{r})|^2 \end{pmatrix}\delta_{\mathbf{r},\mathbf{r}'}\delta_{t,t'}, \\
&\hat{G}^{-1}(\mathbf{r},\mathbf{r}';t-t') = \begin{pmatrix} i\partial_t - \frac{\hat{\mathbf{p}}^2}{2m} + \mu - u(\mathbf{r}) - 2U_0|\psi_0(\mathbf{r})|^2 & -U_0|\psi_0(\mathbf{r})|^2 \\ -U_0|\psi_0(\mathbf{r})|^2 & -i\partial_t - \frac{\hat{\mathbf{p}}^2}{2m} + \mu - u(\mathbf{r}) - 2U_0|\psi_0(\mathbf{r})|^2 \end{pmatrix}\delta_{\mathbf{r},\mathbf{r}'}\delta_{t,t'}.
\end{aligned} \tag{9}$$

After, we can find the formal solution presented in the main text, Eq. (8).
